# Instrument to evaluate the knowledge of patient with cirrhosis on his disease: construction and validity

**DOI:** 10.1186/s12876-021-01665-0

**Published:** 2021-02-23

**Authors:** Mariana Stelmach, Kayo Augusto de Almeida Medeiros, Bárbara Justo Carvalho, Leonardo Zumerkorn Pipek, Gustavo Heluani Antunes de Mesquita, Fernanda Nii, Diego Ramos Martines, Leandro Ryuchi Iuamoto, Luiz Augusto Carneiro D’Albuquerque, Alberto Meyer, Wellington Andraus

**Affiliations:** 1 Faculdade de Medicina FMUSP, Universidade de São Paulo, São Paulo, Brazil; 2 Departamento de Gastroenterologia, Hospital das Clínicas HCFMUSP, Faculdade de Medicina, Universidade de São Paulo, Avenida Doutor Arnaldo, 455, São Paulo, Brazil; 3 Center of Acupuncture, Department of Orthopaedics and Traumatology, University of São Paulo, São Paulo, Brazil

**Keywords:** Liver cirrhosis, Validation studies, Health education, Surveys and questionnaires knowledge

## Abstract

**Background:**

The application of measurement instruments is a strategy to evaluate the patient's knowledge about the disease. This study aims to build an instrument that evaluates the patient's knowledge about liver cirrhosis.

**Method:**

This study includes three phases. The first one was the construction of the instrument based on a literature review. In the second phase, five experts were participated in the evaluation of the instrument, to check the validity of the content. Quantitative and qualitative analyzes were made. The tool used was the CVI (Content Validity Index) and it was used the semantic study of the questions. The third phase was the process of the restructuring the instrument.

**Results:**

The final version of the instrument consisted of 36 questions. The instrument was evaluated in 91.7 by the average CVI and 94.4% by the universal CVI.

**Conclusions:**

The questions are properly structured and clear, therefore, understandable. Thus, the final instrument presented satisfactory content validity, so that, it reached the aim of this study.

## Background

Liver cirrhosis (LC) is a pathological condition that leads to the formation of permanent scars in the liver and impairment of blood flow, which causes loss of its normal function. The main etiological factors are alcohol abuse, viral hepatitis, and autoimmune diseases. In the last decade there was a higher incidence of causes related to cryptogenic disease and non-alcoholic fatty liver disease [1, 2].

In 2018, LC was the 11th main cause of mortality worldwide, responsible for approximately 2 million deaths [1]. Since it is a chronic disease and often related to lifestyle, it requires the patient to change habits, from dietary changes to commitment to medication use and need for continuous care, which can affect quality of life. If we include transplantation to this chronic disease, the only curative therapy, it becomes an even more complex process [3].

It is imperative that health professionals understand the importance of information transmission as a relevant point of their work and develop ways to make information accessible to the population. Low levels of formal education may interfere negatively in the disease prognosis, since the understanding of the medical guidelines is hindered, which may increase mortality rates, visits to emergency centers and hospital readmission [4]. In the case of LC, since it is a chronic disease, it requires changes in lifestyle from the patient, from changes in the diet to commitment to the use of medications and the need for continuous care. That is, there are countless variables that need to be understood by the patient so that the disease does not affect their quality of life. This shows the complexity of factors influencing patients´ adherence. Within this multifaceted context, it becomes fundamental for health professionals to develop strategies to better identify the needs of each patient. From this knowledge, it is possible to perform more tailored interventions, making the patient a protagonist of his own care, and thus, more active in his treatment. Patients who have more information communicate with the healthcare team more effectively and produce better results in their treatments [5].

The patient's understanding of his own physical condition will lead to the development of self-care. In the case of patients who need a solid organ transplant, the need for an educational process becomes even more relevant. In this case, the patient needs to learn how to administer the new medication that he will use for the rest of his life, in addition to adhering to changes in lifestyle, such as hygiene practices, monitoring the new organ, preventing infection and social and professional readjustment. [6].

The use of standard scales as evaluation instruments may contribute to the work of health professionals, helping to track patients in need of follow-up or further intervention. Measurement scales are standardized tools that require a strict process of construction and validation to reach their objectives. When fully validated, they are useful in clinical practice [7].

Some studies have developed and validated instruments to understand the patient's knowledge of his disease. Specifically, in Brazil, we can mention Padilha et. al [8] who investigated beliefs and attitudes in patients with heart valve disease. The work of Bonin et. al [9], built a questionnaire to measure the knowledge of patients with heart failure. The research by Zulianello et. al [10], built and validated a psychometric scale to assess the knowledge of hypertensive patients. In addition, referring to international studies, there is Benhamou et. al [11] who developed and validated an instrument to assess fears and beliefs in patients with knee osteoarthritis; Siklosi et. al [12] who developed and validated a questionnaire to assess patients' knowledge about Cystic Fibrosis; Bardazzi et. al [13] who validated a questionnaire on the awareness of patients with psoriasis; Zschocke et. al [14] who developed a questionnaire to assess adherence factors in patients receiving topical therapy; and Webb et. al [15], who developed an instrument to assess quality of life in patients with primary hyperparathyroidism.

In Brazil, there are some instruments developed for the population of patients with LC. For example, the Chronic Liver Disease Questionnaire (CLDQ) instrument that went through the process of translation and cross-cultural adaptation by Mucci et. al [16], and the Liver Disease Quality of Life (LDQOL), which was also translated and adapted into Portuguese by Teixeira et. al [17]. However, it is worth noting that both instruments aim to assess patients' quality of life.

This study aims to construct and perform the criterion validity of an instrument that is able to assess the knowledge that a patient with cirrhosis has about his disease and its treatment and help health professional have a better understanding of their patients need.

## Methods

### Study design

**Fig. 1:**
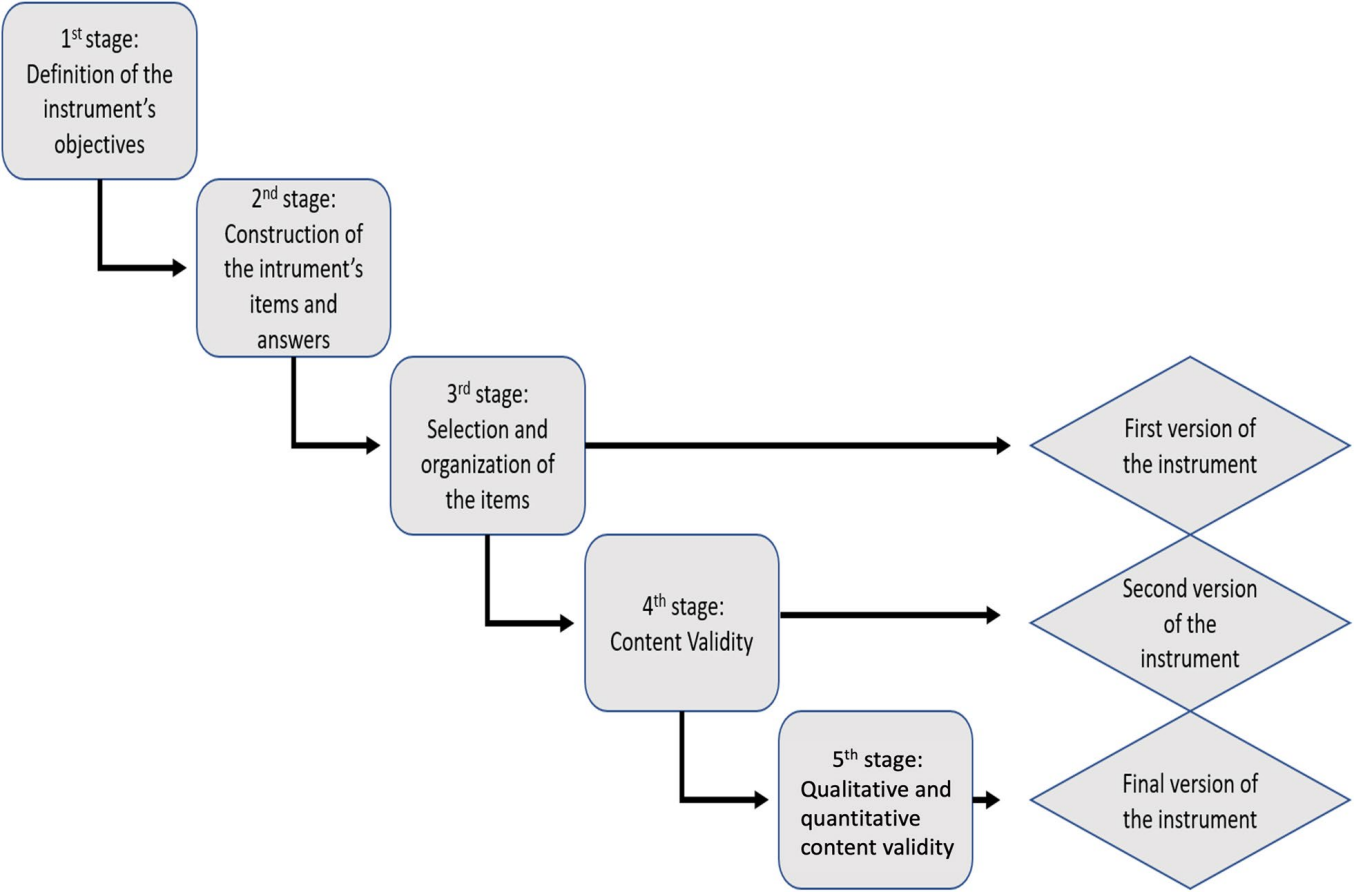
Steps used in the development of the instrument

The development process of the instrument followed the model proposed by Coluci et. al. [18] and can be represented schematically as follows (Fig. 1).

The construction of the instrument´s items (second stage), was carried out based on an extensive literature review, with relevant publications on the theme from the previous five years [18]. The instrument was divided into topics according to a psychometrician for optimization and according to the educational needs of the target population: signs and symptoms, diagnosis, treatment, and medication.

The Likert scale is the most widely used to survey opinions, attitudes, and assessments. In this type of scale, the individual is asked to evaluate the phenomenon (the question) on a 5-point scale. [19, 20] The idea is that the evaluated person chooses for each question presented one of the answer categories Still, according to Gorenstein [21], a Likert scale must strictly have 5 points; any other type of punctuation and / or category must be referred to as “Likert type”. The selection and organization of the items (third stage) should be formulated to present questions according to the principles of clarity, coherence, and neutrality. The drafting of the questions should be done logically to minimize the mental effort of the respondent, using clear and easy to understand language, avoiding complex, ambiguous and excessively long questions [19].

### Data collection

Criterion validity (fourth stage) evaluates the extent to which the test (instrument) measures what it was designed to. In other words, the validity refers to a concordance index between what it measures and what it was designed to measure [22].

Construct and criterion validity was carried out by a committee of specialists. A group of professionals from the Liver Transplant Service Outpatient Clinic of the University of Sao Paulo School of Medicine, was invited to participate in the evaluation, with the requirement that they had at least one year’s experience working with patients with LC. Seven healthcare professionals from different areas were recruited (one in nursing, three doctors, two psychologists, one of them a psychometrician, and a social worker). The literature points to controversies about the number of specialists that should participate in content validation, therefore the minimum parameter cited of five specialists was used, where there was a maximum of ten specialists in the literature [18, 19, 23].

### Data analysis and processing

According to the literature [18], there is no specific statistical test to confirm content validity. Quantitative and qualitative analyses were carried out.

*Quantitative analysis*: the tool used was the Content Validity Index (CVI). The CVI of the question, the mean CVI and the universal CVI were calculated for each topic and for the instrument. The criteria for inclusion of a question were the same proposed by the literature, of 80% agreement between the specialists [18, 19]. The parameters used to calculate each type of CVI are exposed on Table 1.

**Table 1:** Question, mean and universal CVIs calculation summary

	Question CVI	Mean CVI	Universal CVI
Numerator	number of answers "8", "9" or "10"	Summation of all questions' CVIs	Number of questions with CVI > = 80
Denominator	Total number of answers	Total number of questions	Total number of questions
Example	A question was graded by 3 professionals as "8" and by 2 as "6". This question CVI is 60%	If there are five questions, three of them have a CVI of 80% and two of 100%, the mean CVI is 88%	If there are two questions with CVI > = 80 and three with CVI < 80. The universal CVI will be 40%

*Qualitative Analysis*: At the end of each question, the specialists could make suggestions about the content, clarity, and semantics. Additionally, space was left to make general comments and suggest issues or pertinent content that had not been approached.

After the first content validation was performed, a second version of the instrument was made following the specialist’s advices and item’s exclusion criteria. This second version was submitted to a new content validity (stage 5), similar to the first one except for a change in qualitative analysis. Then the instrument would be evaluated based on the CVI of each item in the following criteria: clarity, objectivity, appropriateness for the medical context, for Brazilian culture and for the population evaluated.

## Results

Of the 60 questions that composed the initial version of the instrument, 31 were excluded, of which seven were from the topic “signs and symptoms”, seven from the topic “diagnosis”, eight from the topic “treatment” and nine from the topic “medication”. The exclusion criteria were the question not reaching the CVI of 80% or having repetitive contents in some topics according to the specialists.

Questions were added based on the suggestions of the specialists. The final version of the instrument (in annex 01) had a total of 36 distributed between the topics signs and symptoms, diagnosis, treatment and the subcategory medication. Each topic with 10 and the subcategory with 6 questions.

Questions were formulated based on important information that can help patient make better decision and be aware of the disease, such potential short-term and long-term complications. Common misconceptions, such as that having no symptoms indicates that liver cirrhosis is at the beginning of the disease were also address. Cirrhosis treatment and patient prognosis were also evaluated. The complete questionnaire can be found in supplement material.

### Quantitative analysis—mean and universal CVIs.

Qualitative analysis was based on the mean and universal CVI of each topic and the whole instrument. Information is summarized on Table 2.

**Table 2:** Comparison by topic of the results of mean and Universal CVI in the first and second version of the instrument

		Mean CVI	Universal CVI
First Version	Signs and Symptons	78.7%	67.0%
	Diagnosis	62.6%	53.0%
	Treatment	68.0%	53.0%
	Medication	72.2%	53.0%
	Total	70.4%	56.5%
Second Version	Signs and Symptons	90.0%	90.0%
	Diagnosis	92.0%	90.0%
	Treatment	90.0%	100.0%
	Medication	96.6%	100.0%
	Total	92.2%	95.0%

### Qualitative analysis—instrument semantics

Semantic analysis presented the unanimous suggestion to change the words “hepatic cirrhosis” for “cirrhosis of the liver”. In general, the specialists suggested using simpler and more accessible language for the target population (patients with cirrhosis). The points were pertinent and were followed in the restructuring of the instrument for the second version.

During this analysis, only one question on the “signs and symptoms” topic and on the “medication” topic did not obtain 80% acceptance as seen on Table 3. The remaining questions were included with a CVI of more than 80%, showing that they were assessed as adequate. The specialists did not make new semantic suggestions. There were no changes in the instrument following this analysis, therefore the second and final versions were equal.

**Table 3:** Descriptive analysis of the criteria adopted

Clarity	One question in the topic “signs and symptoms” did not have over 80% acceptance
Objectivity	All questions were adequate
Appropriateness for the medical context	One question in the topic “medication” did not have over 80% acceptance
Appropriateness for Brazilian culture	All questions were adequate
Appropriateness for the target population	All questions were adequate

## Discussion

Liver cirrhosis is a prevalent condition worldwide, with potentially fatal complications and requiring a complex care support network. As a carrier of a severe chronic disease, it is necessary for a patient to undergo various changes in lifestyle, from dietary changes to the use of immunosuppressors. Among the many factors that help patients to adhere to these changes, knowledge about the disease is a variable that can be relayed by the healthcare team.

According to Saberifiroozi [1], the rate of adherence to treatment can greatly increase if the health team is closer, adapting the information to the needs of the patient. Educational groups, continuous contact, and increased capacity for self-management of the patient are examples of actions that the team can perform reflecting on a better prognosis and quality of care.

The creation of an instrument aims to assess the extent of the knowledge that the patient has about the disease. This information can help better understand the patients’ needs aiming to improve adherence and prognosis. The development of the instrument followed the stages described in the literature [18, 19, 24]. Two evaluation approaches were used: one qualitative and one quantitative. The instrument was assessed quantitatively using the CVI. The mean and universal CVIs of each topic were analyzed (signs and symptoms, diagnosis, treatment, and medication). At the end of the evaluation of the first version of the instrument, an average of seven questions were eliminated from each topic for not obtaining the score required for inclusion. This raised the result of the CVI of each topic in the second version of the instrument to a mean higher than 90%, showing an expressive improvement in the results.

The result of the CVI shows that the instrument reached values of concordance among the specialists proposed in the literature. The mean CVI increased from 70.3% to 91.7%. The universal CVI went from 56.7% to 94.4%. Both CVIs obtained over 80% concordance, reaching 90% in the final version, considered ideal by the literature [24, 25]. These results reveal that, with the considerations of the specialists, the instrument was reformulated and significantly improved. Therefore, it achieved satisfactory results for the study proposal.

Similar results were found in other studies using CVI for the evaluation of measuring instruments. An instrument developed about blood transfusion by Bezerra et al. [26] showed a universal CVI of 87%. A study carried out by Fan et al. [27] also aiming to construct instruments for tuberculosis presented a CVI of 96%. Another study focusing on osteoporosis by Rodrigues et al. [28] had a CVI of 87%. These studies showed a convergence in the literature (of around 80%) and presented similar results to the instrument developed in this study, which was 94%.

The qualitative approach was carried out via semantic analysis. The spaces for comments allowed the specialists to express their opinions on each question in a directive and timely manner. This evaluation approach was extremely effective, allowing the transformation of some content into questions from their comments. It is possible to infer that the inclusion of the qualitative analysis allowed the specialists more space and autonomy to express their opinions, making the instrument more appropriate for its purpose. Mostly, the lower rated questions did not use clear language, relying on words that patients would find difficult to understand. Care with the use of clear language is a fundamental requirement for the construction of instruments, as suggested by the literature [29].

The development of an instrument is a complex process that requires the following of several steps to become reliable. One of the largest challenges is that there is no single standard model. We did not find agreement in the literature regarding various issues: the number of questions necessary to make the instrument reliable, the number of specialists to consult, or even a single model to follow for the steps in construction of the model. Each instrument studied had a particular process of development, which required rigorous investigation before choosing the construction stages of this instrument.

We found similar complications regarding the construction of instruments as other authors have previously. For example, the literature highlights the importance of instruments, and warns of the need for a critical evaluation of their psychometric properties. The quality of results that an instrument should aim to reach is only possible with precise and appropriate parameters [24].

In this sense, the need to continue the assessment and psychometric processes of the instrument is reiterated. An important stage was completed: the construction of the instrument. With the completion of the construction of the questions of the instrument, a proposal to be added in later stages is related to the elaboration of the pilot instrument to be applied to the target population [21, 30]. The advantages of including this step are to obtain a more accurate analysis of the understanding of the questions and to evaluate the average time to complete the instrument in the population of patients with cirrhosis. Based on these data acquired with the application, it is possible to make changes to the instrument's structure, regarding the number or semantics of the questions, for example, before developing the final version. Having information about the instrument's performance when using it with the population can make it even more useful and adapted.

## Conclusions

The instrument we developed is a valid tool for assisting healthcare professionals and is an important step towards developing strategies that can contribute to professional healthcare. The greater the knowledge and comprehension of the patient, the more effective the treatment will be, which leads to lower costs for healthcare and better results.

## Supplementary Information

**Additional file 1:** Instrument to evaluate the knowledge of patient with cirrhosis on his disease.

## Data Availability

The datasets used and/or analyzed during the current study are available from the corresponding author on reasonable request.

## Abbreviations

ABTO: Brazilian Association of Organ Transplantation
HCFMUSP: Hospital das Clínicas da Faculdade de Medicina da Universidade de São Paulo
CVI: Content Validity Index
