# Supplementary material for: Instrument to evaluate the knowledge of patient with cirrhosis on his disease: construction and validity
Source: BMC Gastroenterol. 2021 Feb 23;21:83. doi: 10.1186/s12876-021-01665-0 (PMC7903789; doi:10.1186/s12876-021-01665-0)
Supplement: Supplementary file 1 — Additional file 1: Instrument to evaluate the knowledge of patient with cirrhosis on his disease. [file 12876_2021_1665_MOESM1_ESM.docx]

| SIGNS AND SYMPTOMS | |
| --- | --- |
| **Q1** | Malnutrition is responsible for patient’s weakness |
| **Q2** | Itchy skin, yellowing of the skin, ascites, mental confusion (hepatic encephalopathy), esophageal varices are some of the signs and symptoms of liver cirrhosis |
| **Q3** | Having no symptoms indicates that liver cirrhosis is at the beginning of the disease |
| **Q4** | Buildup of water (ascites) is a common complication of liver cirrhosis and is characterized by the accumulation of liquid in the belly |
| **Q5** | Varices of the esophagus (dilatation of the veins of the esophagus) are rare complications of liver cirrhosis |
| **Q6** | Liver cirrhosis, in some cases, presents complications (esophageal varices, mental confusion, ascites) that should be treated and accompanied by a doctor to avoid worsening the patient’s health situation. |
| **Q7** | Tiredness is not a complication of liver cirrhosis, tiredness is caused by major depression |
| **Q8** | Liver Encephalopathy presents: mental confusion, disorientation, hand shakes, slurred speech, slow movements, memory loss and in more serious cases can lead to coma. |
| **Q9** | A cirrhotic liver releases toxic substances that can attack the heart and cause heart attacks |
| **Q10** | Patients who suffer attacks hepatic encephalopathy with signs of mental confusion, disorientation, slow movement, etc. should always be accompanied when leaving the house |

| DIAGNOSIS | |
| --- | --- |
| **Q1** | Cirrhosis is characterized by lesions on the liver that make the organ lose its functions |
| **Q2** | The only cause of liver cirrhosis is consumption of alcohol |
| **Q3** | It is possible to have cirrhosis of the liver and hepatocarcinoma (liver cancer) at the same time |
| **Q4** | Liver cirrhosis requires continuous medical surveillance to evaluate the evolution of the illness |
| **Q5** | Liver cirrhosis can be brought about by different causes, for example: hepatitis B or C, alcohol, fatty liver and autoimmune diseases |
| **Q6** | Liver cirrhosis is a contagious disease transmitted by mosquito bites |
| **Q7** | Often the disease is discovered when the patient presents complications of the disease: ascites, mental confusion (encephalopathy), esophageal varices, yellowing of the skin |
| **Q8** | Liver cirrhosis allows moderate consumption of alcoholic drinks, in other words it is possible to drink and can of beer, a glass of wine or a measure of spirits per day. |
| **Q9** | Liver cirrhosis is considered a chronic disease, in other words it will follow the individual for life. |
| **Q10** | A higher MELD score indicates a greater seriousness of the disease |

| TREATMENT | |
| --- | --- |
| **Q1** | The treatment of liver cirrhosis only needs medical surveillance |
| **Q2** | When transplantation is indicated as a treatment for liver cirrhosis, professional medical follow up is needed (doctors, nurses, psychologists, nutritionists, social workers), both in pre-transplantation and post-transplantation. |
| **Q3** | Orientations of nutritionists about diet are not indicated in the treatment of liver cirrhosis, as there is no food that can aggravate the symptoms and the disease |
| **Q4** | Liver transplantation is a surgery in which the sick liver (with cirrhosis) is substituted for a healthy liver |
| **Q5** | When entering the waiting list for liver transplantation the patient must undergo assessments and enter into regular follow up with a number of professionals such as nurses, nutritionists, psychologists, social workers, etc. |
| **Q6** | The treatment of liver cirrhosis requires a network of social support, in other words, help from friends and/or families for hospital trips, use of medication and daily tasks |
| **Q7** | After entry to the waiting list for liver transplantation, the wait for an organ can be stressful. During this time symptoms of anxiety and depression can appear, needing treatment from psychologists and psychiatrists |
| **Q8** | To receive adequate care after transplantation surgery the patient stays in the hospital for some time, and in serious cases or complications may go to the ICU |
| **Q9** | After recovery from transplant surgery it is no longer necessary to be in follow up, considering that the illness was cured |
| **Q10** | The patient may have different emotional reactions (fears, anxieties, sadness, worry) during the treatment of liver cirrhosis, in the pre- and post- transplant periods and because of this psychological treatment is important |

| MEDICATION | |
| --- | --- |
| **Q1** | The medication used to treat liver cirrhosis cause sexual impotence |
| **Q2** | The medications are not to cure liver cirrhosis but to control the symptoms of the disease |
| **Q3** | It is important that the medications are taken according to medical advice, following doses and hours |
| **Q4** | The transplantation aims to cure liver cirrhosis, meaning that after having it, it is no longer necessary to take any medication |
| **Q5** | Following drug treatment can be helped by family support, who can help with the time and correct use of medication, for example. |
| **Q6** | After the transplantation the patient should use medication to avoid rejection (immunosuppressor drugs) for the rest of their lives |

**Original Version**

| SINAIS E SINTOMAS | |
| --- | --- |
| **Q1** | Os sintomas da cirrose hepática são curados pela mudança dos hábitos de vida |
| **Q2** | Não ter sintomas significa que a cirrose hepática não é considerada grave |
| **Q3** | Coceiras pelo corpo, amarelão, ascite e encefalopatia, são alguns sintomas da cirrose hepática |
| **Q4** | A cirrose hepática pode causar desnutrição deixando o paciente fraco |
| **Q5** | Os pacientes podem ter o diagnóstico de cirrose e não apresentar sintomas, ou seja, ter a doença compensada |
| **Q6** | A ascite é uma complicação comum da cirrose e caracteriza-se pela retenção de líquido na barriga |
| **Q7** | A cirrose hepática, em alguns casos, apresenta complicações (varizes de esôfago, encefalopatia, ascite) que devem ser tratadas pois podem complicar ainda mais o estado de saúde |
| **Q8** | Seguir as orientações do nutricionista sobre a alimentação podem auxiliar no controle da ascite e encefalopatia complicações comuns na cirrose hepática |
| **Q9** | A encefalopatia hepática apresenta como sintomas: confusão mental, desorientação, tremores nas mãos, fala arrastada, movimento lento, e em casos mais graves pode levar ao coma |
| **Q10** | A cirrose hepática pode desencadear sintomas de ansiedade e depressão |
| **Q11** | A encefalopatia hepática pode afetar a sua habilidade de trabalhar e dirigir podendo provocar riscos de acidentes de carro |
| **Q12** | Ao apresentar sintomas de icterícia, ou seja, o amarelão, significa que o fígado está se regenerando e apresentando sinais de melhora |
| **Q13** | Os pacientes que apresentam crises de encefalopatia hepática devem ser acompanhados ao sair de casa |
| **Q14** | O fígado com cirrose libera substâncias tóxicas que podem atacar o sistema cardiovascular provocando uma parada cardíaca |
| **Q15** | O paciente ao apresentar crise de encefalopatia hepática deve procurar ajuda considerando que o quadro pode piorar rapidamente e se tornar uma condição de emergência |

| DIAGNÓSTICO | |
| --- | --- |
| **Q1** | A cirrose hepática é uma doença que ataca o fígado |
| **Q2** | A cirrose hepática pode ser ocasionada por diferentes causas, por exemplo, hepatites, álcool e doenças autoimunes, entre outras causas |
| **Q3** | A cirrose hepática é uma doença que acomete pessoas idosas devido ao enfraquecimento do funcionamento do fígado |
| **Q4** | O diagnóstico de cirrose hepática não exige uma alteração nos hábitos alimentares |
| **Q5** | A cirrose hepática é considerada uma doença grave e se não tratada pode levar a morte |
| **Q6** | A cirrose hepática possibilita o consumo moderado de álcool |
| **Q7** | A biópsia no fígado é um dos exames utilizados para o diagnóstico de cirrose hepática |
| **Q8** | O diagnóstico de cirrose hepática proíbe a prática de exercícios físicos praticados pois podem prejudicar o funcionamento do fígado |
| **Q9** | A única causa da cirrose hepática é o consumo exagerado de álcool |
| **Q10** | A cirrose hepática é uma doença contagiosa |
| **Q11** | A cirrose hepática é considerada uma doença crônica, ou seja, irá acompanhar o indivíduo ao longo da vida |
| **Q12** | O fígado doente na cirrose hepática pode prejudicar o funcionamento de outros órgãos |
| **Q13** | O diagnóstico de cirrose hepática torna fundamental o acompanhamento médico contínuo para detectar precocemente possíveis complicações da doença |
| **Q14** | A cirrose hepática é ocasionada pelo exagero de bebidas quentes ingeridas diariamente |
| **Q15** | O hepatocarcinoma (câncer no fígado) é uma doença que pode estar presente junto com o diagnóstico de cirrose hepática |

| TRATAMENTO | |
| --- | --- |
| **Q1** | Em casos graves de cirrose a única forma de tratamento é o transplante |
| **Q2** | O transplante caracteriza-se pela troca de um fígado doente por outro sadio |
| **Q3** | O transplante é considerado uma cirurgia de baixa complexidade |
| **Q4** | Alguns alimentos podem prejudicar o paciente com cirrose hepática e precisam ser evitados, por isso é necessário um acompanhamento com nutricionista |
| **Q5** | Ao ter o diagnóstico de cirrose hepática é necessário se organizar com uma rede de suporte social para auxiliar com as idas ao hospital, uso de medicação e cuidados durante o tratamento |
| **Q6** | Ao entrar na fila de transplante é necessário seguir em acompanhamento multiprofissional (enfermagem, nutrição, psicologia, serviço social) |
| **Q7** | A fila de transplante funciona de acordo com a ordem de chegada, ou seja, os pacientes inscritos por primeiro recebem o órgão antes do segundo, e assim consecutivamente |
| **Q8** | Pode ocorrer rejeição do novo órgão após o transplante, e isso significa a possibilidade de um retransplante |
| **Q9** | Ao entrar na fila de transplante, inicia-se um período de espera pelo órgão. Esse período pode ser bastante estressante, levando a desenvolver sintomas de ansiedade e depressão que devem ser acompanhados por psiquiatras e psicólogos |
| **Q10** | Após o transplante não será mais necessário acompanhamento médico ou ir ao hospital pois a doença estará curada |
| **Q11** | Os hábitos de vida (trabalho, atividades físicas e alimentação) necessitam ser adaptados após o transplante |
| **Q12** | Para receber os cuidados adequados, após a cirurgia de transplante o paciente vai para UTI e posteriormente para enfermaria |
| **Q13** | O MELD do paciente é um dos critérios para alocação na fila de transplante (resultado de um exame) |
| **Q14** | Uma vez inscrito na fila de transplante não tem mais como ser retirado |
| **Q15** | Um valor elevado de MELD indica uma maior gravidade da doença |

| MEDICAMENTOS | |
| --- | --- |
| **Q1** | O uso continuo de medicamentos pode causar outros prejuízos (ter efeito negativo) para a saúde |
| **Q2** | Os medicamentos usados para tratar a cirrose hepática causam impotência sexual |
| **Q3** | As medicações prescritas pelo médico são em quantidade exagerada e provocam efeitos colaterais indesejados |
| **Q4** | A lactulona é um dos principais medicamentos utilizados no tratamento da encefalopatia hepática |
| **Q5** | Os medicamentos prescritos pelo médico não são para curar a cirrose mas para tratar e controlar os sintomas da doença |
| **Q6** | Antes de tomar algum medicamento é fundamental conversar com o médico pois alguns medicamentos devem ser evitados por prejudicar ainda mais o fígado doente |
| **Q7** | O tratamento com medicamentos pode curar a cirrose hepática e não precisar mais do transplante |
| **Q8** | É importante que os medicamentos sejam ingeridos de acordo com a orientação médica, seguindo a dosagem exata e os horários |
| **Q9** | Após o transplante devo fazer uso da medicação (drogas imunossupressoras) pelo resto de vida |
| **Q10** | Os medicamentos imunossupressores baixam a imunidade do organismo o que leva a uma maior probabilidade de adquirir outras doenças (resfriados, por exemplo), por isso, é necessário ter um maior cuidado com a saúde |
| **Q11** | O transplante tem como objetivo a cura da doença do fígado, o que significa que após realizá-lo não será mais necessário usar nenhum medicamento |
| **Q12** | É possível ficar sem tomar os medicamentos imunossupressores até no máximo uma semana sem causar prejuízos ao novo órgão transplantado |
| **Q13** | Ao apresentar um efeito colateral indesejado dos imunossupressores devo informar o médico antes de fazer uso de qualquer medicamento |
| **Q14** | Os medicamentos imunossupressores são muito caros o que dificulta muitas vezes utilizá-los conforme orientação médica |
| **Q15** | A adesão ao tratamento medicamentoso pode ser facilitada com o apoio da família, que pode auxiliar com o horário e o uso correto do medicamento, por exemplo. |

| **Final Version** | |
| --- | --- |
| SINAIS E SINTOMAS | |
| **Q1** | A cirrose no fígado pode causar desnutrição e deixar o paciente fraco |
| **Q2** | Coceiras pelo corpo, amarelão na pele, barriga d`água, confusão mental (encefalopatia hepática), varizes de esôfago são alguns sinais e sintomas da cirrose no fígado |
| **Q3** | Não ter sintomas significa que cirrose no fígado está no inicio da doença |
| **Q4** | A barriga d`água (ascite) é uma complicação comum da cirrose no fígado e caracteriza-se pelo acúmulo de líquido na barriga |
| **Q5** | As varizes de esôfago (dilatação das veias do esófago) são complicações raras nos casos de cirrose no fígado |
| **Q6** | A cirrose no fígado, em alguns casos, apresenta complicações (varizes de esôfago, confusão mental, barriga d`água) que devem ser tratadas e acompanhada pelo médico para evitar o agravamento do estado de saúde |
| **Q7** | O sintoma de cansaço não é uma complicação da cirrose no fígado, o cansaço é causado por um quadro de depressão grave |
| **Q8** | A encefalopatia hepática apresenta como sinais: confusão mental, desorientação, tremores nas mãos, fala arrastada, movimentos lentos, perda de memória, e em casos mais graves pode levar ao coma |
| **Q9** | O fígado com cirrose libera substâncias tóxicas que podem atacar o coração e provocar uma parada cardíaca |
| **Q10** | Os pacientes que apresentam crises de encefalopatia hepática como sinais de confusão mental, desorientação, movimentos lentos, etc, devem sempre sair de casa acompanhados |

| DIAGNÓSTICO | |
| --- | --- |
| **Q1** | A cirrose caracteriza-se por lesões no fígado que fazem com que o órgão perca as suas funções |
| **Q2** | A única causa da cirrose no fígado é o consumo de bebida alcoólica |
| **Q3** | É possível ter cirrose no fígado e hepatocarcinoma (câncer no fígado) ao mesmo tempo |
| **Q4** | A cirrose no fígado exige acompanhamento médico contínuo para avaliar a evolução da doença |
| **Q5** | A cirrose no fígado pode ser ocasionada por diferentes causas, por exemplo: hepatite B ou C, álcool, gordura no fígado e doenças autoimunes |
| **Q6** | A cirrose no fígado é uma doença contagiosa transmitida pela picada de um mosquito |
| **Q7** | Muitas vezes a doença de cirrose no fígado é descoberta quando o paciente apresenta complicações da doença: barriga d'àgua, confusão mental (encefalopatia), varizes de esôfago, amarelão na pele |
| **Q8** | A cirrose no fígado permite o consumo moderado de bebida alcoólica, ou seja é possível beber uma lata de cerveja, uma taça de vinho ou uma dose de destilado por dia. |
| **Q9** | A cirrose no fígado é considerada uma doença crônica, ou seja, irá acompanhar o indivíduo ao longo da vida |
| **Q10** | Um valor elevado de MELD indica uma maior gravidade da doença |

| TRATAMENTO | |
| --- | --- |
| **Q1** | O tratamento da cirrose no fígado exige somente acompanhamento médico |
| **Q2** | Quando o transplante é indicado como tratamento para a cirrose no fígado é necessário ter acompanhamento dos profissionais (médicos, enfermeiros, psicólogos, nutricionistas, assistente social) tanto no período de pré-transplante quanto no pós-transplante. |
| **Q3** | Orientações dos nutricionistas sobre alimentação não são indicadas no tratamento da cirrose no fígado, já que nenhum alimento pode agravar os sintomas e a doença |
| **Q4** | O transplante no fígado é uma cirurgia em que o fígado doente (com cirrose) é substituído por um fígado sadio |
| **Q5** | Ao entrar na lista de transplante do fígado é necessário passar por avalições e seguir em acompanhamento com diversos profissionais, como enfermeiros (as), nutricionistas, psicólogo (as), assistentes sociais, etc. |
| **Q6** | O tratamento da cirrose no fígado exige uma rede de suporte social, ou seja, ajuda de amigos e/ou familiares para idas ao hospital, uso de medicação e tarefas da vida diária |
| **Q7** | Após a inscrição na lista de transplante do fígado a espera pelo órgão pode ser estressante, neste período pode surgir de sintomas de ansiedade e depressão que precisam ser acompanhados por psiquiatras e psicólogos |
| **Q8** | Para receber os cuidados adequados após a cirurgia de transplante no fígado o paciente fica um período internado na enfermaria, e somente em casos graves ou complicações pode ir para a UTI |
| **Q9** | Após a recuperação da cirurgia de transplante do fígado não é mais necessário acompanhamento médico considerando que a doença foi curada |
| **Q10** | O paciente pode ter diferentes reações emocionais (medos, angustias, tristezas, inquietações) durante o tratamento da cirrose no fígado, no período pré e pós-transplante por isso é importante o acompanhamento psicológico |

| MEDICAMENTOS | |
| --- | --- |
| **Q1** | Os medicamentos usados para tratar a cirrose no fígado causam impotência sexual |
| **Q2** | Os medicamentos não são para curar a cirrose no fígado mas para controlar os sintomas da doença |
| **Q3** | É importante que os medicamentos sejam tomados de acordo com a orientação médica, seguindo as dosagens e os horários |
| **Q4** | O transplante tem como objetivo a cura da cirrose do fígado, o que significa que após realizá-lo não será mais necessário usar nenhum medicamento |
| **Q5** | A adesão ao tratamento medicamentoso pode ser facilitada com o apoio da família, que pode auxiliar com o horário e o uso correto do medicamento, por exemplo. |
| **Q6** | Após o transplante devo fazer uso da medicação contra a rejeição (drogas imunossupressoras) pelo resto de vida |
